# In vitro efficacy of different PEGylation designs on cathelicidin-like peptide with high antibacterial and antifungal activity

**DOI:** 10.1038/s41598-023-38449-3

**Published:** 2023-07-11

**Authors:** Seray Sahsuvar, Tanil Kocagoz, Ozgul Gok, Ozge Can

**Affiliations:** 1 Department of Medical Biotechnology, Institute of Health Sciences, Acibadem Mehmet Ali Aydinlar University, Istanbul, Turkey; 2 Department of Biomedical Engineering, Faculty of Engineering and Natural Sciences, Acibadem Mehmet Ali Aydinlar University, Istanbul, Turkey; 3 Department of Medical Microbiology, School of Medicine, Acibadem Mehmet Ali Aydinlar University, Istanbul, Turkey

**Keywords:** Microbiology, Biochemistry, Chemical modification, Peptides, Nanobiotechnology, Peptide delivery

## Abstract

Recent reports on antibiotic resistance have highlighted the need to reduce the impact of this global health issue through urgent prevention and control. The World Health Organization currently considers antibiotic resistance as one of the most dangerous threats to global health. Therefore, Antimicrobial peptides (AMPs) are promising for the development of novel antibiotic molecules due to their high antimicrobial effects, non-inducing antimicrobial resistance (AMR) properties, and broad spectrum. Hence, in this study, we developed novel antimicrobial peptide/polymer conjugates to reduce the adverse effects of TN6 (RLLRLLLRLLR) peptide. We demonstrate how our constructs function in vitro in terms of antimicrobial activity, hemolytic activity, cytotoxicity, and protease resistance. Our findings show that our molecules are effective against different types of microorganisms such as *Staphylococcus aureus*, *Escherichia coli*, *Pseudomonas aeruginosa*, methicillin-resistant *S. aureus*, vancomycin-resistant *Enteroccus faecium*, and *Candida albicans*, which are known to be pathogenic and antibiotic-resistant. Our constructs generally showed low cytotoxicity relative to the peptide in HaCaT and 3T3 cells. Especially these structures are very successful in terms of hemotoxicity. In the bacteremia model with *S. aureus*, the naked peptide (TN6) was hemotoxic even at 1 µg/mL, while the hemotoxicity of the conjugates was considerably lower than the peptide. Remarkably in this model, the hemolytic activity of PepC-PEG-pepC conjugate decreased 15-fold from 2.36 to 31.12 µg/mL compared to the bacteria-free 60-min treatment. This is proof that in the case of bacteremia and sepsis, the conjugates specifically direct to bacterial cell membranes rather than red blood cells. In addition, the PepC-PEG-pepC conjugate is resistant to plasma proteases. Moreover, morphological and intracellular damage of the peptide/conjugates to *Escherichia coli* are demonstrated in SEM and TEM images. These results suggest our molecules can be considered potential next-generation broad-spectrum antibiotic molecule/drug candidates that might be used in clinical cases such as bacteremia and sepsis.

## Introduction

Antimicrobial resistance (AMR), which results from the continuous and unnecessary use of antibiotics, is a present threat to public health^1,2^. Bacteria become resistant to a new antibiotic molecule approximately in 10 years^3^. While this process is natural, AMR gradually increases with new pathogenic bacteria because of antibiotics misuse and climate change^4^. Instead of conventional antibiotics, there is a need to discover antimicrobial drugs with an innovative mechanism of action that does not trigger or possibly minimizes AMR. One of the novel antibiotics against AMR will be antimicrobial peptides (AMPs).

Most AMPs are the promising drugs of the future because they have a rapid, broad-spectrum mechanism of action and do not induce AMR^5^. After the introduction of gramicidin and polymyxin B in 1955, which were the first Food and Drug Administration (FDA)–approved AMPs, approval of other AMPs has decelerated over the years, with very few AMPs passing clinical trials. AMPs are helper molecules produced by microorganisms to protect themselves from competitors or hosts in nature. AMPs have short amphiphilic residue (12–50), a cationic net charge and most of them have an α-helical structure^6^. Cathelicidins are cationic peptides with amphipathic properties that are found among natural AMPs^7^. The mechanism of cathelicidin to kill microorganisms is very similar with other antimicrobial peptides.

Some adverse effects of the AMPs, such as toxicity to host cells, quick renal clearance, enzyme sensitivity, immunogenicity, and short plasma half-life, have limited their use in clinical practice^8^. Recently, AMPs, proteins, lipids, and other molecules have been conjugated with polymers such as Polyethylene glycol (PEG) to mitigate their side effects. PEG is a polyether composed of repeating ethylene glycol units of varying molecular weights. PEGylation is a modification of molecules by covalent conjugation strategies with PEG polymer, which itself is non-immunogenic and non-toxic^9^. The peptide distribution, conjugation site, and structure of PEG may significantly affect the toxicity, solubility, and antimicrobial activity of a drug candidate^10^. However, information about how linear PEG location and length influence the antimicrobial activity and toxicity of AMPs in vitro and in vivo has been scant^11^.

Therefore, this study formed novel PEG-AMP conjugates in different structures using TN6 peptide (RLLRLLLRLLR), a cathelicidin-like helical peptide with high antimicrobial activity^12^. Here we demonstrate how the binding of single or paired TN6 AMP to a linear PEG affects in vitro experiments. We also present the effect of in vitro hemolytic, cytotoxicity, protease resistance and antimicrobial properties of molecular constructs produced by PEG bound to AMPs in different orientations.

## Results

### Synthesis, purification, and characterization of PEG-AMP conjugates

**Figure 1:**
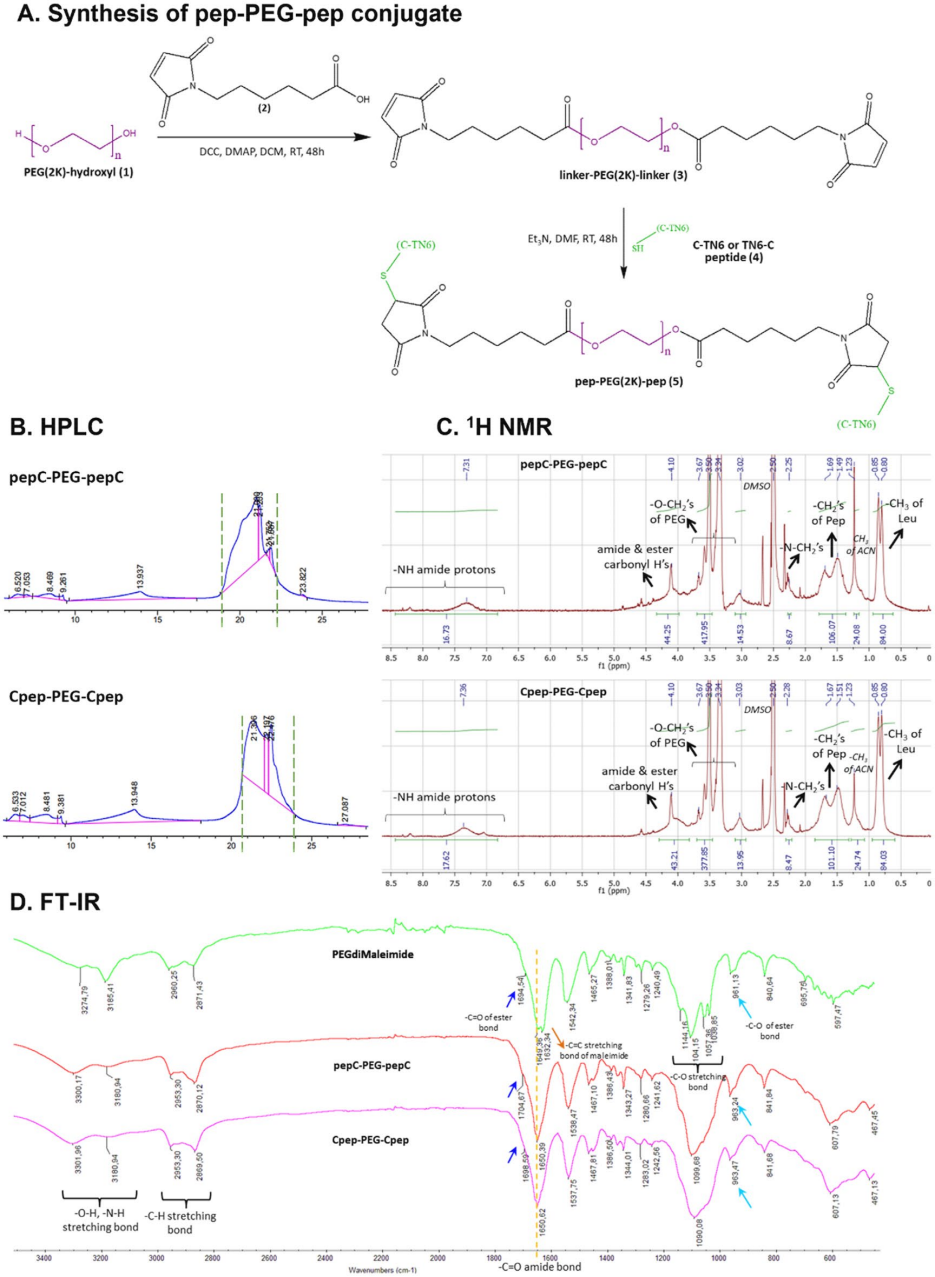
Synthesis and characterization of pep-PEG-pep conjugates. (**A**) Schematic illustration of stepwise synthesis of pep-PEG-pep conjugates. (**B**) HPLC chromatograms of pep-PEG-pep conjugates. (**C**) ^1^H NMR profile of the pep-PEG-pep conjugates revealing the characteristic protons from the peptide and PEG molecule. (**D**) FT-IR profile of PEGdiMaleimide and pep-PEG-pep conjugates to verify intermolecular bonding.

**Figure 2:**
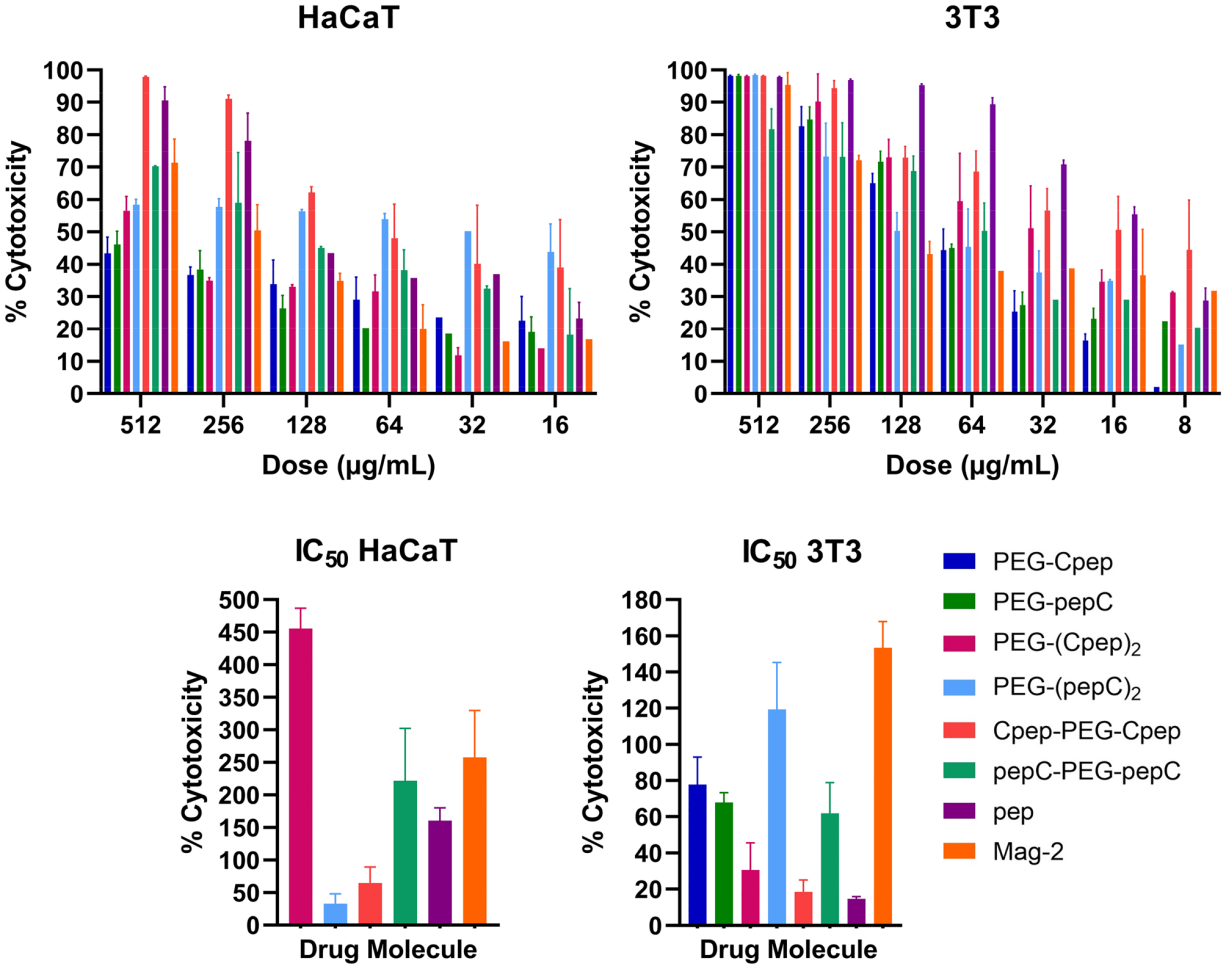
Dose-dependent cytotoxic effect of conjugates against human skin keratinocyte (HaCaT) and mouse embryonic fibroblast (3T3) cells for 24 h. Cytotoxic effects of the synthesized peptide/conjugate, which are PEG-Cpep, PEG-pepC, PEG-(Cpep)_2_, PEG-(pepC)_2_, Cpep-PEG-Cpep and pepC-PEG-pepC, pep and Mag-2 were tested on HaCaT and 3T3 cell lines. Mag-2 was used in the cytotoxicity test for AMP-based conjugates as a control group. The inhibitory concentration (IC_50_) value was calculated using the MTT assay formulation (Methods). Data were expressed as mean ± SD (three replicates) and subjected to two-way analysis of variance (ANOVA). P values of graphs were considered statistically significant (p < 0.0001).

**Figure 3:**
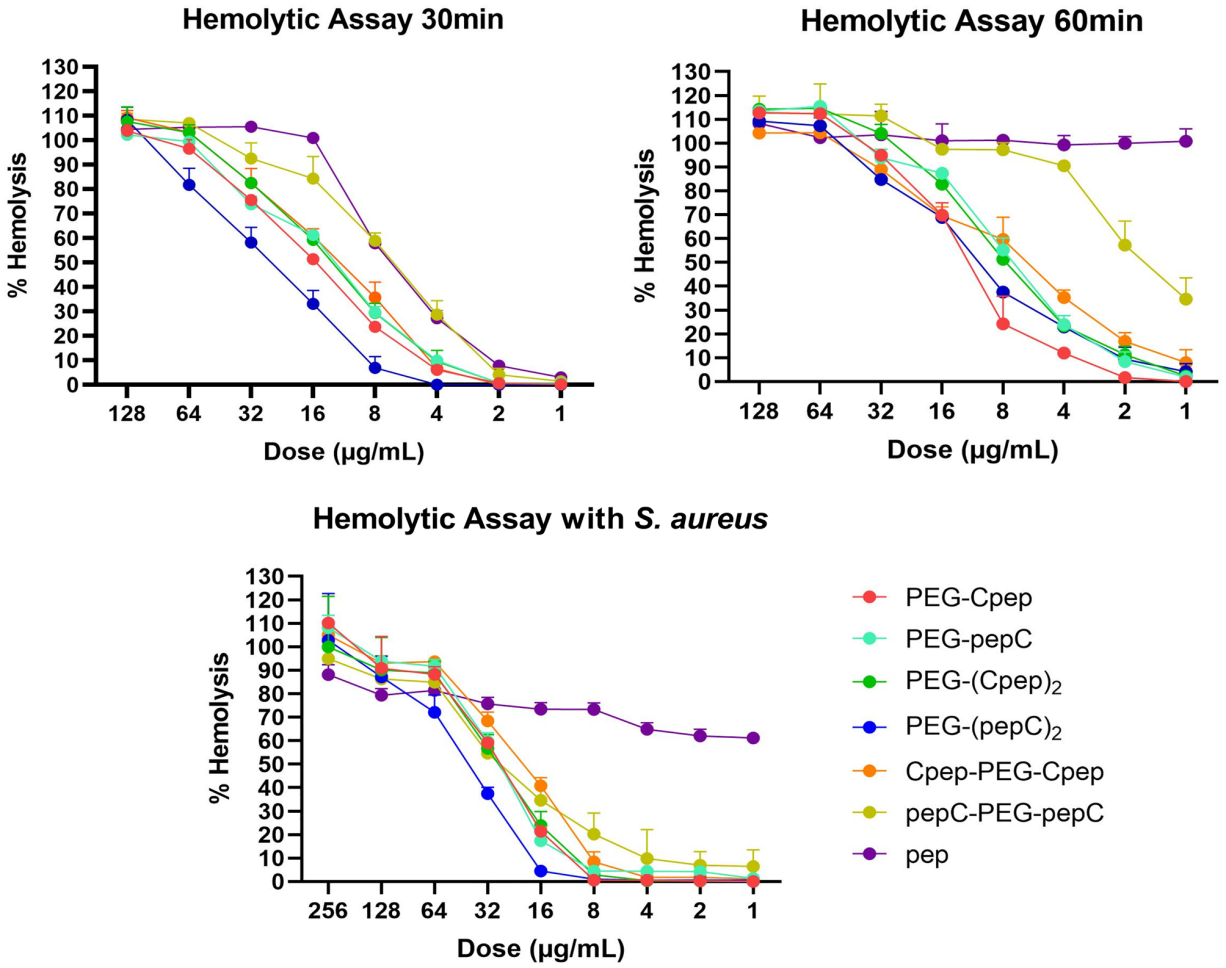
Dose-dependent hemolytic activity assay of peptide/conjugate against human erythrocytes. The hemolytic effects of all peptide/conjugate were tested on human erythrocytes at 30 min, 60 min, and 60 min (with *S. aureus* ATCC 25923). Hemolytic assay data were expressed as mean ± SD (three replicates) and subjected to two-way ANOVA. P values of graphs are statistically significant (p < 0.0001).

Peptides where cysteine (C) is located at the N-terminal or C-terminal have been synthesized to modify them. The sequence and physical properties of the synthesized modified peptides are given in Table 1 (Supp. Data 1, Fig. 1A). These features are the same for both C-TN6 and TN6-C. Figure 1 summarizes the synthesis, purification, and analysis results of the pep-PEG-pep conjugate from the PEG-AMP designs. Supp. Data 1 (Figs. 1A–E, 2A–D, 3A–D) presents the analysis results for the other conjugate designs and peptide molecules. The peptide-polymer conjugates prepared in this study vary not only in peptide numbers but also in architecture. To synthesize the conjugate, a functional PEG molecule and peptide were linked using different heterofunctional linkers. Before binding with the PEG polymer, peptides were analyzed using LC–MS/MS (Supp. Data 1, Fig. 1C). Fragments were determined after peptide ionization using molecular weight/charge (m/z) ratio in Table 2 (Supp. Data 1, Fig. 1B) from the + ESI scan diagram. In the LC–MS/MS spectrum, only the peptide fragment peaks and the solution peak (113–114) were obtained. For the pep-PEG-pep conjugate synthesis (Fig. 1A), the PEGdiMaleimide molecule was prepared by reacting PEG(2K) hydroxyl (1) with 6-Maleimidohexanoic acid linker (2) in the presence of coupling agents to obtain a linker-PEG(2K)-linker (3) molecule with maleimide ends. The PEGdiMaleimide molecule was further reacted with the C-TN6 or TN6-C peptide (4) using thiol-maleimide reaction (1,4-conjugate addition reaction) to generate the pep-PEG-pep conjugate (6). The conjugates were purified using HPLC, and the peaks between the green dashed lines were collected (Fig. 1B). In both pep-PEG-pep conjugates, conjugate peaks were obtained between 21 and 22 min, corresponding to a buffer composition of 67–70% ACN. Since the pep-PEG-pep conjugate has two hydrophobic peptide molecules, it also caused the total conjugate to elute within the hydrophobic range during purification. When the HPLC chromatograms of the conjugates were compared, the hydrophobicity of the pep-PEG-pep conjugate was increased compared with PEG-(pep)_2_ since the PEG molecule was embedded within the pep-PEG-pep structure, leaving no free PEG end in the total structure. When PEG-pep and PEG-(pep)_2_ conjugates were compared, the HPLC chromatograms (elution at 10% ACN) were almost identical because only one side of the PEG molecule was masked even though the peptide count in the conjugates was different (Supp. Data 1, Figs. 2A, 3A). In addition, the purity of all conjugates collected after HPLC is above 95% (data not shown). The chemical structures of these conjugates were examined using proton NMR spectroscopy (Fig. 1C), taken in d_6_-DMSO, which could clearly demonstrate both peptide-specific protons and those representing the PEG polymer part. The conjugates prepared by either the N-terminus-modified peptide (C-TN6) or the C-terminus-modified one (TN6-C) were expected to produce similar protons in their spectra (Supp. Data 1, Fig. 1D). However, the difference in the integration of peptide-specific isopropyl -CH_3_ protons comes from the doubled amount of peptide conjugated to the same molecular weight polymer. A comparative representation of the spectra for peptides indicates the presence of protons belonging to both labile ones appearing at around 6.9–8.7 ppm, amide carbonyl protons between amino acids as a broad multiple at 4.10 ppm and, a doublet at 0.84 ppm with a coupling constant *J* = 20 for isopropyl groups of the leucine amino acid. For all conjugates (PEG-pep, PEG-(pep)_2_, and pep-PEG-pep), it is possible to see those peptide peaks along with the broad and high-intensity triplet at around 3.50 ppm representing –O-CH_2_ protons of PEG chains (Fig. 1C, Supp. Data 1, Figs. 2C, 3C). By comparing the NMR of a single peptide-containing conjugate (PEG-pep) (Supp. Data 1, Fig. 2C) with two peptide-containing ones, it can easily be understood that not only the integration of isopropyl groups but also those for amide carbonyl peaks have almost doubled, confirming the desired ratio of polymer to peptide in conjugate structures, originating from the functional group types and amounts in heterofunctional linker molecules. Meanwhile, the differentiation between the bivalent peptide-containing conjugate PEG-(pep)_2_ (Supp. Data 1, Fig. 3C) and the bipeptidic pep-PEG-pep (Fig. 1C) conjugate could be raised from the extra ester carbonyl peaks at the latter’s spectra at 3.67–3.25 ppm because of the protons in the structure of the heterofunctional linker molecule. FT-IR analysis of the conjugates showed the presence of the bonds formed by the PEG molecule and peptides. O–H and N–H stretching bonds (3302–3180 cm^−1^), C–H stretching bond (2870–2960 cm^−1^), and C=O amide bond (~ 1650 cm^−1^) were seen at the same wavenumbers for the peptide (Supp. Data 1, Fig. 1E), pep-PEG-pep (Fig. 1D) and all other conjugates (Supp. Data 1, Figs. 2D, 3D). Furthermore, C–O stretching bond (1060–1145 cm^−1^) from the PEG molecule was demonstrated in all conjugates. The PEGdiMal molecule, which was used as an intermediate for preparation the pep-PEG-pep conjugate, has a C=C bond in the maleimide group with a sharp stretching peak at 1632 cm^−1^. The disappearance of the C=C stretching bond suggests that the peptide molecules have been conjugated to the linker on both sides. In addition, C-O of the ester group (~ 960 cm^−1^) and C=O of (1695–1705 cm^−1^) the ester group in the linker structure was observed in the pep-PEG-pep conjugate.

**Table 1:** MICs for the peptide/conjugate against various microorganisms.

Microorganism	Minimum Inhibitory Concentration (µg/mL)							
	PEG-Cpep	PEG-pepC	PEG-(Cpep)_2_	PEG-(pepC)_2_	Cpep-PEG-Cpep	pepC-PEG-pepC	TN6	Ampicillin
*S. aureus* ATCC 25923	64	16	128	64	4	32	1	8
*E. coli* ATCC 25922	64	16	128	128	4	64	1	32
*P. aeruginosa* ATCC 27853	128	128	256	256	32	128	4	256
*C. albicans* ATCC 10231	32	32	128	64	8	16	1	16
*MRSA* ATCC *33591*	84	21	120	105	12	25	0.78	32
*VRE-faecium* ATCC *BAA-2316*	84	21	120	52	12	25	1.56	16

**Table 2:** Stability of the TN6 peptide and pep-PEG-pep conjugates against plasma proteases.

Hours (h)	MIC after plasma proteases (µg/mL)			
	Pep	Cpep-PEG-Cpep	pepC-PEG-pepC	Blood plasma
0	2.56	36.6	36.6	None
6	2.56	18.3	36.6	None
24	5.12	18.3	36.6	None

### in vitro evaluation of polymer-peptide conjugates

In all in vitro cell experiments, concentrations were calculated by considering the mass of the whole conjugate, not per peptide domain.

The peptide/conjugate and control group (ampicillin) were tested on six different pathogenic species. These species are *Staphylococcus aureus* (*S. aureus*) and *Escherichia coli* (*E. coli*), which are common Gram-positive and negative bacteria, respectively; *Pseudomonas aeruginosa* (*P. aeruginosa*) and methicillin-resistant *S. aureus* (*MRSA*)/vancomycin-resistant *Enteroccus faecium* (*VRE-faecium*)*,* which are common Gram-negative and Gram-positive hospital bacteria, respectively; and *Candida albicans* (*C. albicans*), a pathogenic yeast. All conjugate designs were tested with microorganisms with different properties of resistance, Gram type, and shape, and they were characterized by differing antimicrobial/antifungal activities. The MICs of the peptide/conjugate on these species were determined. The same results were obtained for all replicates. The PEG molecule or the linkers used alone have not exhibited any antimicrobial activity, while the TN6 peptide has shown high antimicrobial activity. Considering the MICs results, the Cpep-PEG-Cpep conjugate demonstrated the highest antimicrobial/antifungal activity compared to ampicillin and all other conjugates in all tested microorganisms, while the PEG-(Cpep)_2_ conjugate showed the lowest activity. Except for *C. albicans* ATCC 10231, the PEG-pepC conjugate demonstrated the second-best inhibitory effect. The pepC-PEG-pepC conjugate revealed the second-best activity on *C. albicans* ATCC 10231 (Table 1).

The MTT assay was carried out to evaluate the in vitro cytotoxic effect of all conjugates. The cytotoxicity effect was found to be dependent on conjugate concentration. Cell viability declined with increasing conjugate concentrations. Mag-2 is a broad-spectrum natural peptide with well-known antimicrobial activity. Due to these properties, it was used as a control group in the cytotoxicity test^13^ (Fig. 2).

The inhibitory concentration (IC_50_) values of the HaCaT cells were > 512 µg/mL, > 512 µg/mL, 455.63 µg/mL, 33.22 µg/mL, 64.81 µg/mL, 222.02 µg/mL, 160.55 µg/mL, and 257.67 µg/mL for PEG-Cpep, PEG-pepC, PEG-(Cpep)_2_, PEG-(pepC)_2_, Cpep-PEG-Cpep, pepC-PEG-pepC, pep, and Mag-2, respectively (Fig. 2). In the HaCaT cell line, the IC_50_ values were compared. PEG-(Cpep)_2_ exhibited the lowest cytotoxic activity, while PEG-(pepC)_2_ showed the highest cytotoxic effect. This major difference in the PEG-(pep)_2_ conjugate design is probably due to the difference in the peptide end whose side is attached to the PEG. No IC_50_ dose was detected even at the maximum concentration (512 µg/mL) tested in PEG-pep conjugates. In pep-PEG-pep conjugates, meanwhile, pepC-PEG-pepC has a significantly less cytotoxic effect than the Cpep-PEG-Cpep conjugate. PEG-(pepC)_2_ and Cpep-PEG-Cpep conjugates which included two peptides, were more toxic than the peptide itself.

In the 3T3 cell line, IC_50_ values were 77.80 µg/mL, 67.87 µg/mL, 30.55 µg/mL, 119.26 µg/mL, 18.35 µg/mL, 61.84 µg/mL, 14.56 µg/mL, and 153.31 µg/mL for PEG-Cpep, PEG-pepC, PEG-(Cpep)_2_, PEG-(pepC)_2_, Cpep-PEG-Cpep, pepC-PEG-pepC, pep, and Mag-2, respectively (Fig. 2). According to IC_50_ values, the least cytotoxic conjugate was PEG-(pepC)_2_, while the most cytotoxic was Cpep-PEG-Cpep. The peptide was the most toxic molecule at all tested concentrations compared to all samples.

To understand the in vitro hemotoxic effect of all conjugates, the concentration-dependent percentages of hemolysis were determined at different time points in Fig. 3. The TN6 peptide showed the highest hemolytic activity at all time points. After 30 min incubation, all doses tested for the TN6 peptide were well above the toxic dose (HC_50_), which killed 50% of the red blood cells.

For the 30 min hemolytic assay, among the conjugates treated with fresh human erythrocytes, PEG-(pepC)_2_ had the lowest hemolysis rate at all concentrations, while pepC-PEG-pepC had the highest hemolysis rate. Hemolytic activity of the TN6 peptide and all conjugates increased at 60 min of treatment compared to 30 min. At 60 min, while the HC_50_ values of the PEG-(pepC)_2_ and pepC-PEG-pepC conjugates were tripled (60 min HC_50_: 10.38 µg/mL and 2.36 µg/mL, respectively), hemolytic activity was almost doubled in PEG-pepC, PEG-(Cpep)_2_, and Cpep-PEG-Cpep conjugates (60 min HC_50_ 7.23 µg/mL, 7.71 µg/mL, and 6.84 µg/mL, respectively). The lowest increase in hemolytic activity was seen in PEG-Cpep (60 min HC_50_: 12.73 µg/mL).

For the 60 min treatment, the cell-associated hemolytic assay with *S.aureus* ATCC 25923 revealed the lowest hemolytic activity of PEG-(pepC)_2_ (HC_50_: 49.60 µg/mL), while the highest was Cpep-PEG-Cpep (HC_50_: 24.48 µg/mL). The cell-associated hemolytic activity of pepC-PEG-pepC significantly decreased 15-fold from 2.36 µg/mL to 31.12 µg/mL compared with the 60 min treatment without bacteria. Hemolytic HC_50_ doses of PEG-pepC, PEG-(Cpep)_2_, and Cpep-PEG-Cpep declined approximately 4 times from 7.23 µg/mL, 7.71 µg/mL, and 6.84 µg/mL (60 min) to 29.44 µg/mL, 30.98 µg/mL, and 24.48 µg/mL (60 min with bacteria), respectively. A 2.5-fold reduction in hemolytic activity (from 12.73 to 31.28 µg/mL) was also observed in PEG-Cpep. The naked peptide (TN6) showed a hemotoxic effect at all doses in hemolysis data with or without bacteria in 60 min.

To examine the effect of human plasma proteases on the antimicrobial activities of the peptide/conjugate samples, the MIC values of samples were determined after incubation with human plasma. After sampling at 0-, 6-, and 24-h intervals, they were treated with bacteria as specified in the procedure.

The design (pep-PEG-pep) containing Cpep-PEG-Cpep with the best MIC value at *S. aureus*, *E. coli*, *P. aeruginosa*, *C. albicans*, MRSA, VRE-*faecium* was selected as a candidate for stability assay toward plasma proteases. The blood plasma used as a negative control group showed no antimicrobial activity against *S. aureus* 25923, which is one of the most common bacteria causing bacteremia^14^.

The peptide’s antimicrobial activity declined from 2.56 to 5.12 µg/mL after 24 h. When Cpep-PEG-Cpep and pepC-PEG-pepC were compared, MIC values that were the same at 0 h (36.6 µg/mL) remained the same at all time intervals for pepC-PEG-pepC, while the MIC value for Cpep-PEG-Cpep decreased to 18.3 µg/mL, the latter indicating better antimicrobial activity than before. The same results were obtained in all replicates (Table 2).

**Figure 4:**
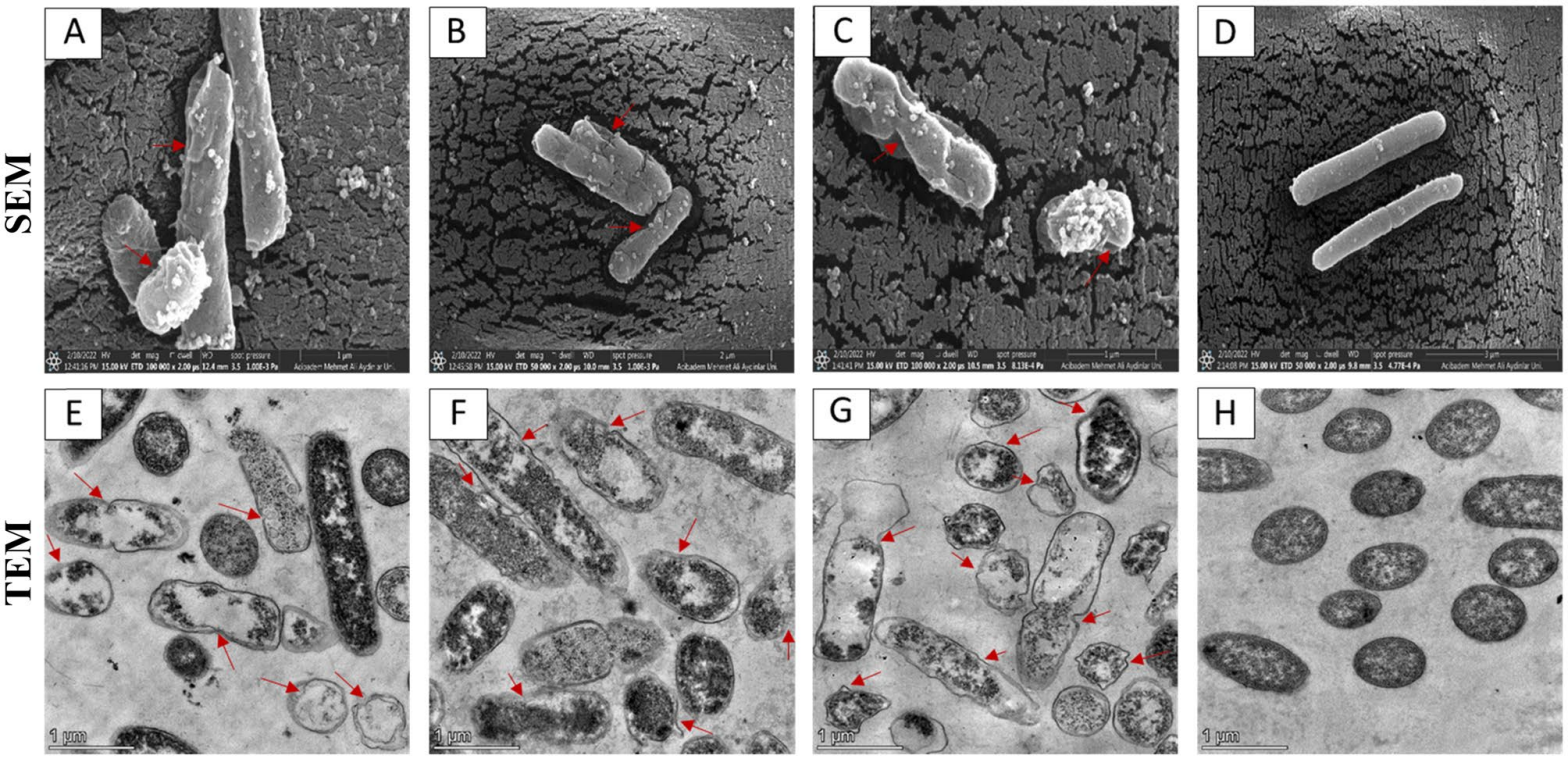
The effect of Cpep-PEG-Cpep (**A**–**E**), pepC-PEG-pepC (**B**–**F**) conjugates and TN6 (**C**–**G**) antimicrobial peptide on *E. coli* were observed using scanning electron microscopy (SEM) transmission electron microscopy (TEM). *E. coli* ATCC 25922 (**D**–**H**) was used as a control group. In *E. coli*, the peptides, because of their positively charged amino acids (arginine), attracted the negatively charged phosphate groups of the bacteria’s membrane lipids, shrinking the bacterial membrane, and reducing its thickness. The peptide/conjugate also causes the internal cellular components to be released by creating pores and channel structures.

SEM was used to visualize the pep-PEG-pep conjugates and TN6 peptide-treated *E. coli* ATCC 25922 cellular damage. Control cells that were untreated with conjugates or peptides exhibited smooth surfaces (Fig. 4D), but conjugate- and peptide-treated cells showed morphological changes (Fig. 4A–C). Specifically, the regions marked with red arrows indicate the change in membrane shape. The membrane surfaces of the *E. coli* cells became rough and indented after peptide/conjugate treatment. Cpep-PEG-Cpep (Fig. 4A) and pepC-PEG-pepC (Fig. 4B) exhibited similar damage patterns on bacterial membranes.

After pep-PEG-pep conjugate and TN6 peptide treatment, intracellular changes in *E. coli* and differentiations of the bacterial cell wall were observed using TEM. As a control group, *E. coli* showed homogeneous cytoplasm and complete bacterial membrane (Fig. 4H). In contrast, the peptide/conjugate displayed significant cell wall and membrane disruption, bacterial cell content release, and obvious cytoplasmic clear zones (Fig. 4E–G). In addition, most *E. coli* cells had heterogeneous cytoplasm. Cpep-PEG-Cpep (Fig. 4E) and pepC-PEG-pepC (Fig. 4F) showed the same cellular damage mechanisms. Presumably, when the conjugates enter the cell by forming pores, intracellular contents are released.

## Discussion

For millions of years, various organisms have used AMPs for the self-defense^15^. AMPs have become promising next-generation antibiotics because of their high antimicrobial effect, non-inducing AMR property, and broad-spectrum. The number of synthetic AMPs designed to mimic nature has increased in recent years. For instance, new AMPs produced through the creation of synthetic derivatives of cathelicidins have shown much higher antimicrobial activity than cathelicidin itself^12^. However, only a small number of AMPs have received FDA approval because of their high toxicity, short plasma half-life, low stability and poor solubility^16^.

In this study, followed a PEGylation strategy to minimize the possible side effects of AMPs. For this purpose, six different novel PEG-AMP conjugates were developed to understand the effect of parameters such as the conjugation site of the PEG polymer, peptide orientation and the effect of the number of bound AMPs. TN6 peptide, a synthetic derivative of cathelicidin, was used in the developed conjugates because of its high antimicrobial activity. TN6 peptides were linked to the PEG polymer through their N- or C-termini via a terminal cysteine residue. The antimicrobial activity, protease activity, toxicity, and stability of all conjugates were systematically evaluated in vitro.

We investigated the potential of our conjugates to become candidates for next-generation broad-spectrum antibiotics by testing PEG-AMP conjugates against different types of microorganisms. The PEG polymers used were non-toxic, highly soluble molecules^17^ and did not show antimicrobial activity (data not shown). The naked TN6 peptide exhibited the highest antimicrobial activity compared with the conjugates. One may speculate that an AMP attached to the PEG may show reduced activity because of the steric hindrance created by the surrounding PEG branches. Other studies observed that the antimicrobial activity of natural^18^ or synthetic^19^ AMPs with α-helical structure decreased after PEGylation. The reduction in antimicrobial activity can be more drastic when the active end of the AMP is masked. To understand this phenomenon, AMPs were attached from either end to the PEG polymer and evaluated. The results helped us understand which side of the peptide is more active depending on its orientation within the whole structure. Interestingly, the TN6 peptide conjugated via its N-terminus in the pep-PEG-pep design has shown the highest inhibitory effect. This would normally be regarded as the C-terminus of the TN6 peptide being required to be unattached to a structure to exhibit its activity. However, in the PEG-pep and PEG-(pep)_2_ conjugate designs, peptide conjugation via the C-terminus exhibited superior antimicrobial activity for most of the tested microorganisms (Table 1). Therefore, no generalization can be made regarding the active terminus of the AMPs in AMP-containing conjugates. This can be explained by the major difference in the conjugate designs. For the pep-PEG-pep conjugate structure, there is less possibility of the polymer being able to sterically hinder the free ends of the peptide. In this architecture, where the peptides are attached to the polymer from its different sides, the peptide is given more flexibility during its entry into the bacteria. Meanwhile, when the PEG polymer and the two peptides are attached via one branched, trifunctional linker as in the PEG-(pep)_2_ design, antimicrobial activity is reduced. This may be due to the steric hindrance caused by the free PEG polymer on one side of the peptide and the possibility of two peptides suppressing each other’s activity through non-covalent interactions. We have observed similar peptide peptides in molecular dynamics studies; that is when peptides aggregate or stay close to each other, the activity of the molecules is always reduced whereas when they are free to approach the membrane, they exert better antimicrobial activity (data not shown).

The cytotoxicity of the conjugates on mammalian cells were determined, and their MIC values were compared. The cytotoxicity of TN6 peptide was significantly higher than those of most conjugates in 3T3 and HaCaT cell lines. This indicates that the polymer–peptide conjugates reduced cytotoxicity towards the naked peptide. This may be because the hydrophilic PEG polymer forms a stable structure by preventing the aggregation of its peptide, and the polymer prevents non-specific cell and protein binding by surrounding the peptide^20,21^.

When the IC_50_ values of the conjugates tested in the HaCaT and 3T3 cell lines were compared, all conjugates except PEG-(pepC)_2_ showed significantly lower cytotoxicity in the HaCaT cell line. Interestingly, the PEG-(pepC)_2_ conjugate showed the highest cytotoxicity in the HaCaT cell line but the lowest cytotoxicity in the 3T3 cell line. Therefore, only the PEG-(pepC)_2_ conjugate exerts cytotoxicity in the HaCaT cell line based on the MIC values of conjugates. In addition, conjugates with the PEG-pep design did not exceed the IC_50_ in all concentrations studied. In the 3T3 cell line, MIC values of the PEG-Cpep, PEG-pepC, Cpep-PEG-Cpep, and pepC-PEG-pepC conjugates did not exceed the IC_50_ cytotoxicity value (within safe ranges indicated by error bars) in all tested bacteria except *P. aeruginosa*. The PEG-(pepC)_2_ conjugate did not exhibit cytotoxicity for the MIC values of all bacteria except *E. coli* and *P. aeruginosa*. Meanwhile, all MIC values tested in the PEG-(Cpep)_2_ conjugate were toxic to the 3T3 cell line (Fig. 2).

Keratinocytes and dermal fibroblasts represent the main cell type of the epidermis and dermis, respectively. The HaCaT (keratinocyte) cell line is found in all epidermis layers, while the 3T3 (fibroblast) cell line is found in the dermis^22,23^. The reason why all the molecules except PEG-(pepC)_2_ show more toxic effects in the 3T3 cell lines can be explained by the higher tendency of keratinocytes to collect in cell clusters on surfaces as in the skin layer^24,25^, and in this case, their physical resistance may be higher. Also, past studies have shown that antimicrobial agents cause more cytotoxic effects in the 3T3 than the HaCaT cell line^26^. In future in vivo experiments, conjugates are not expected to encounter fibroblasts at desired doses (i.e., concentrations between MIC and IC_50_ cytotoxicity values) as in in vitro experiments. Therefore, most of the conjugate molecules will first reach the keratinocytes and will act in this region in the case of topical application, such as ointments. Thus, the toxic effects of the conjugates, if any, are expected to be lower for fibroblasts than the associated values obtained in in vitro experiments.

To evaluate the in vitro hemolytic effect of the peptide/conjugate, the percentage of concentration-dependent hemolysis was determined by treating compounds with human erythrocytes at different time intervals. The TN6 peptide exhibited the highest hemolytic activity at all time intervals. After 30 min of incubation, all doses tested for the TN6 peptide were shown as hemotoxic. The hemolytic activity of the peptide/conjugate increased at 60 min of treatment compared to 30 min. However, this increase made naked TN6 peptide toxic even at 1 µg/mL. Introducing the PEG polymer in the structure significantly reduced the hemolytic effect (Fig. 3).

Bacteremia is a clinical condition characterized by the presence of bacteria in the bloodstream^27^. When *S. aureus* was added to the 60 min hemolytic activity test setup, a significant decline was observed in the hemolytic activities of all conjugates. The PEG-(pepC)_2_ conjugate (HC_50_: 49.60 µg/mL) had the lowest hemolytic activity at 60 min with bacteria. However, the naked TN6 peptide continued to show a hemotoxic effect even with addition of bacteria. We speculate that with the addition of bacteria, the peptide/conjugate preferentially targets the bacterial membrane rather than the erythrocyte membrane, thereby diminishing hemolytic activity. Likewise, the addition of bacteria resulted in a 15-fold decrease in the hemolytic activity of the pepC-PEG-pepC conjugate at 60 min. Therefore, the conjugates might be effective antibiotic candidates in future clinical cases such as bacteremia or sepsis.

When MIC, cytotoxicity and hemolytic results are evaluated, the pep-PEG-pep design stands out. Considering all the conjugates, the pep-PEG-pep design, in which the polymer could not hinder the end groups of the peptide molecules, gave the best MIC results. In cytotoxicity results, the effect of Cpep-PEG-Cpep and pepC-PEG-pepC conjugates in both 3T3 and HaCaT cell lines are correlated with MIC values. Accordingly, the pepC-PEG-pepC conjugate has a significantly less cytotoxic effect than the Cpep-PEG-Cpep conjugate. According to the results of *S. aureus*-associated hemolytic activity together with MIC values, it was shown that HC_50_ values were not exceeded when MIC values of pep-PEG-pep conjugates for *S. aureus* were used as drug dose when applied to red blood cells. Also, the significant change in this experiment (from 60 min hemolytic activity to 60 min *S. aureus*-associated hemolytic activity) was a 15-fold decrease in the hemolytic activity of the pepC-PEG-pepC conjugate. This result shows us that this design is particularly successful to targeting the bacterial membrane. Hence, we decided that the pep-PEG-pep conjugate was the most advantageous design overall.

After treatment with plasma proteases, the MIC values of the peptide/conjugate were investigated at different time points. As expected, the MIC value for the TN6 peptide increased. The value for the Cpep-PEG-Cpep conjugate decreased, while that for the pepC-PEG-pepC conjugate remained constant (Table 2). The reason why the antimicrobial activity of the Cpep-PEG-Cpep conjugate increases over time might be that the proteases damage the C-TN6 peptide attached to this conjugate, causing its fragmentation. Accordingly, the new peptide sequence formed with the remaining amino acids after cleavage still has a certain length, amphiphilic characteristics, and a positive charge. It might have increased its antimicrobial activity because of these properties. The results show that the resistance of the peptide to plasma proteases clearly increases during the PEGylation process.

Likewise, the TN6 peptide, a cathelicidin-like AMP, uses pattern similar to that of cathelicidins in killing microorganisms. In Fig. 4, positively charged peptide/conjugate interacts with the negatively charged cell membranes of microorganisms to form transmembrane pores that directly kill the microorganisms^28,29^. Peptide/conjugate also forms a hydrophilic channel between the charged groups, allowing water molecules and other hydrophilic molecules to move^12^. As clearly seen in the SEM/TEM results, the cell membrane has become rough and the cell cytoplasm heterogeneous because of the transmembrane pores formed by the peptides/conjugate. In addition, intracellular compartments were observed to leak out of the membrane (Fig. 4).

## Conclusion

In conclusion, the constructs developed in this study successfully demonstrated in vitro antimicrobial activity against six different microorganism types. These properties and their low adverse effects highlight PEG-AMP conjugates as potential next-generation broad-spectrum antibiotics. Moreover, they can be considered promising candidates for addressing the lack of suitable drugs against various pathogen-resistant microorganisms.

## Materials

### Chemical synthesis and purification/analysis

1-[3-(dimethylamino)propyl]-3-ethylcarbodiimide methiodide (EDC), N,N′-dicyclohexylcarbodiimide (DCC), 4(dimethylamino)pyridine (DMAP), triethylamine (Et_3_N), anhydrous dimethylformamide (DMF), diisopropylcarbodiimide (DIC), anhydrous N-Hydroxysuccinimide (NHS), trifluoroacetic acid (TFA), Oxyma®, piperidine, dichloromethane (DCM), acetonitrile (ACN), toluene, amyl acetate, ethyl alcohol, propylene and uranyl acetate were purchased as American Chemical Society (ACS) grade from Sigma-Aldrich, USA.

### In vitro experiments

Mueller–Hinton agar (MHA, Oxoid), Mueller–Hinton broth (MHB, Oxoid), dimethyl sulfoxide (DMSO, Sigma-Aldrich), Triton X-100 (Sigma Aldrich), fetal bovine serum (FBS, ATCC 30-2020™), penicillin–streptomycin (Pen/Strep, Gibco™), Dulbecco’s modified Eagle’s medium (DMEM, Gibco™), and protease inhibitor (cOmplete™ Mini Protease Inhibitor Cocktail, Roche) were used.

### Cells

*Staphylococcus aureus* (*S. aureus* ATCC 25923), *Escherichia coli* (*E. coli* ATCC 25922), *Pseudomonas aeruginosa* (*P. aeruginosa* ATCC 27853), *Candida albicans* (*C. albicans* ATCC 10231), methicillin-resistant *S. aureus* (*MRSA*, ATCC 33591), and vancomycin-resistant *Enteroccus faecium* (*VRE-faecium*, ATCC BAA-2316) as bacterial strains; human skin keratinocyte (HaCaT, HEKa, PCS-200-011™, ATCC) and mouse embryonic fibroblast (3T3, CRL-1658™, ATCC) as mammalian cells were used.

### Methods

#### Peptide synthesis and analysis

Peptides given in Table 1 (Supp. Data 1, Fig. 1A) were synthesized using a peptide synthesizer (CEM, Liberty™ Blue and CEM Discover™, USA) following standard Fmoc protocol on Rink amide resin (0.7 mmol/g loading capacity), which was used to synthesize amide end peptides from the C-terminus. The synthesis scale was determined as 0.10 mmol. Before synthesis, the resin was kept in DMF solvent for 30 min to swell. All amino acids used 0.2 M (in DMF) and L-form conformation. Amino acids and resin have protecting groups to prevent undesirable side reactions during synthesis. In the initial step of the synthesis cycle (resin loading), the first amino acid was anchored to the resin from the C-terminal after the resin’s functional deprotection group was removed. The anchored peptide is extended by repetitive cycles which include deprotection and coupling steps. The experiment used 20% piperidine as deprotection and 1.0 M Oxyma and 0.5 M DIC as the activator base and activator for coupling steps, respectively. After the last N-terminal deprotection, the peptides were cleaved from the resin using peptide cleavage system (CEM, Razor™, USA). The resin was washed three times with DCM, after which 5 mL of TFA/triisopropylsilane/ddH_2_O (95:2.5:2.5, v/v/v) mixture was added, and incubated for 35 min at 38 °C^30^. The crude peptides were precipitated three times in cold diethyl ether (− 20 °C) and centrifuged at 4500 rpm for 3 min, dried under vacuum, and purified by RP-HPLC (260 Infinity Quaternary Liquid Chromatography systems, Agilent Technologies) C-18 column (RPC 250 × 10 mm ID hydrophobic 6 µm, Agilent VariTide) using an appropriate 5–80% ACN (0.025% TFA)/ddH_2_O (0.05% TFA) gradient. 6420 Triple Quad LC/MS (Agilent Technologies, USA) was used to determine peptide mass and MS2scan method was used. Samples were analyzed for 1 min at 135 V for 500–2200 Da.

#### Preparation of polymer-peptide conjugates

Three main conjugate designs were used, PEG-pep, PEG-(pep)_2_ and pep-PEG-pep, and each design was separately synthesized with two different peptides, Cpep (C-TN6) and pepC (TN6-C). All synthesis reactions were performed under anhydrous conditions and nitrogen gas.

For the PEG-pep conjugate synthesis (Supp. Data 1, Fig. 2A), Mal-PEG_6_-NHS ester (2) (5 mg, 0.0083 mmol, Sigma Aldrich) linker and C-TN6 or TN6-C peptide (1) (14.05 mg, 0.0091 mmol) in 1 mL DMF were added to a round-bottom flask (rbf), followed by the addition of Et_3_N (2.3 µL, 0.0207 mmol) at room temperature (RT). After 2 h, methoxy PEG(2K)-NH_2_ (4) (19.12 mg, 0.0095 mmol, Sigma-Aldrich) in 1 mL DMF was added to the reaction mixture (3) and stirred for 48 h at RT. Then, the mixture (5) was precipitated in cold diethyl ether overnight (– 20 °C).

For the PEG-(pep)_2_ conjugate synthesis (Supp. Data 1, Fig. 3A), in the first step, the carboxylic acid end of the Bis-Mal-Lysine-PEG_4_-acid (2) (20 mg, 0.0287 mmol, BroadPharm) linker was activated by NHS (3.97 mg, 0.0345 mmol) and EDC (8.27 mg, 0.0431 mmol) in 1 ml DMF and mixed in a rbf on a magnetic stirrer at RT. After 24 h, the NHS-activated linker was precipitated in cold diethyl ether (– 20 °C) for 12 h and centrifuged at 5500 rpm for 10 min and left to dry under high vacuum. In the second step, NHS-activated linker (3 mg, 0.0037 mmol), C-TN6 or TN6-C peptide (1) (12.21 mg, 0.0079 mmol), and Et_3_N (1 µL, 0.0076 mmol) were added to the new rbf at RT. After 2 h, methoxy PEG(2K)-NH_2_ (4) (9.46 mg, 0.0047 mmol, Sigma Aldrich) in 1 mL DMF was added to the reaction (3) and continued for 2 days at RT. Then, the reaction (5) was precipitated in cold diethyl ether overnight (– 20 °C).

For the pep-PEG-pep conjugate synthesis (Fig. 1A), in the first step, the production of PEG-Linker (PEGdiMal) was synthesized. The PEG polymer was dried using azeotropic distillation with toluene. PEG(2K)-OH (200 mg, Sigma Aldrich) dissolved in 50 mL toluene. Toluene was then removed using an evaporator and left to dry under high vacuum. The dried PEG(2K)-OH (1) (200 mg, 0.1 mmol), 6-Maleimidohexanoic acid linker (2) (50.7 mg, 0.24 mmol, Sigma Aldrich), DMAP (14.7 mg, 0.12 mmol), DCC (54.5 mg, 0.264 mmol), and 10 mL anhydrous DCM were mixed in a rbf on a magnetic stirrer at RT for 48 h. After the reaction, dichloromethane was removed using an evaporator and the remaining solid was dissolved in 20 mL ethyl acetate. The solution was then filtered to remove DCU which is converted from DCC. Ethyl acetate from the filtered sample was evaporated and dissolved in DCM. Then, the mixture was precipitated in cold diethyl ether overnight (– 20 °C). The remaining solids were washed with DCM again and filtered. DCM was removed using an evaporator and left in a high vacuum overnight. In the second step, C-TN6 or TN6-C peptide (4) (20.64 mg, 0.0134 mmol), PEGdiMal (3) (13 mg, 0.0054 mmol) molecule and Et_3_N (1.5 µL, 0.0107 mmol) were mixed in 1.2 mL anhydrous DMF and left on a magnetic stirrer. After 48 h, the mixture (5) was precipitated in cold diethyl ether overnight (– 20 °C).

#### Conjugate purification and characterization

The conjugates were purified using RP-HPLC C-18 column for the pep-PEG-pep conjugate, 0–100% (ACN/ddH_2_O gradient) for 30 min, and for the PEG-pep and PEG-(pep)_2_ conjugates, 1–10% for 7 min, 10–100% for 1 s, 100–100% for 2 min. Chemical characterization of peptide/conjugate was measured using Fourier transform infrared spectroscopy (FT-IR, Nicolet iS10 Thermo Fisher) to determine the presence of the functional groups. The chemical structure of the purified peptide/conjugate was characterized using proton nuclear resonance (^1^H NMR) on a 400 MHz Bruker NMR spectrometer in DMSO-d_6_.

#### Minimum inhibitory concentration

*Staphylococcus aureus* (*S. aureus* ATCC 25923), *Escherichia coli* (*E. coli* ATCC 25922), *Pseudomonas aeruginosa* (*P. aeruginosa* ATCC 27853), *Candida albicans* (*C. albicans* ATCC 10231), methicillin-resistant *S. aureus* (MRSA, ATCC 33591) and vancomycin-resistant *Enteroccus faecium* (VRE-*faecium,* ATCC BAA-2316) were grown in MHA at 37 °C, overnight. A colony was then selected and transferred to MHB. Each bacterial solution in the MHB was adjusted to 0.5 McFarland with OD600 value between 0.08 and 0.1 using UV–Vis spectrophotometer (NanoDrop™ One/OneC Microvolume, Thermo Scientific). Afterward, 0.5 McFarland bacteria cultures were diluted in MHB at a ratio of 1:100. The peptide/conjugate concentration was quantified using a UV–Vis spectrophotometer at 214 nm. Serial dilutions of peptide and conjugates (1024–0.5 µg/mL) were prepared in MHB with a total volume of 95 µL in a round-bottom 96-well plate. Of the prepared bacterial suspensions, 5 µL was added into sample dilutions in each prepared well. Ampicillin (GeneMark) was used as a positive control and bacterial solution alone was used as a negative control. All 96-well plates were incubated overnight at 37 °C, and all samples were prepared in triplicate. Then, samples were measured at 600 nm using a microplate reader (Gen5 Synergy HT BioTek, USA)^31^.

#### Cytotoxicity assay

Human skin keratinocyte (HaCaT, HEKa, PCS-200-011™, ATCC) and mouse embryonic fibroblast (3T3, CRL-1658™, ATCC) cells were counted to 5 × 10^4^ cells/well in 100 µL of DMEM containing 10% FBS, and 1% Pen/Strep antibiotics were placed in a flat-bottom 96-well plate and incubated in 5% CO_2_ at 37 °C. After 24 h, the medium on the attached cells was discarded. Serial peptide/conjugate dilutions (1024–0.5 µg/mL) prepared in complete DMEM (100 µL of total volume) in triplicate were added to the cell-seeded plates and incubated in 5% CO_2_ at 37 °C for 24 h. Cytotoxicity was determined using the 3-(4, 5-dimethylthiazol-2-yl)-2, 5-diphenyltetrazolium bromide assay kit (MTT, Cell Proliferation Kit, Roche) following the kit procedure and measured at 550 nm and 690 nm using a microplate reader. Cytotoxicity was calculated according to the cell control samples in complete DMEM.

#### Hemolytic activity assay

Human blood cells provided by healthy donors were used for this study. The study was performed depending on the Helsinki Declaration, and approval was provided by Acibadem Mehmet Ali Aydinlar University Medical Research Ethics Committee (No 2023-2/32; 27 January 2023). Written informed consent was provided from all donors before draw blood.

300 µL of fresh human blood (Acibadem Mehmet Ali Aydinlar University, Istanbul, Turkey) was suspended in 20 mL of sterile tris-saline (150 mM NaCl, 10 mM Tris, pH 7.2) solution and centrifuged 3 times at 1500 rpm for 5 min. Pellet was dissolved with 100 mL of tris-saline and 100 µL blood mix was added into each well of a round-bottom 96-well plate. Serial peptide/conjugate dilutions (1024–0.5 µg/mL) prepared in tris-saline (100 µL of total volume) in triplicate were added on top of the blood mix. 10% DMSO prepared in Triton X-100 treated with blood mix used as a positive control, and the blood mix itself was used as a negative control. The samples were kept on separate round-bottom 96-well plates at 37 °C for 30 min and 1 h, respectively, to examine two different conditions. After incubations, the 96-well plates were centrifuged at 1500 rpm for 10 min. The supernatants were then transferred into a new round-bottom 96-well plate for measurement at 414 nm using a microplate reader^31^. The lysis percentage of the peptide/conjugate was calculated using Eq. (1) as follows:

1 $$\mathrm{Hemolysis } \, \left(\mathrm{\%}\right)=\frac{\mathrm{Sample } \, \left(414 \, \mathrm{ nm}\right)-\mathrm{Negative \, control }\left(414 \, \mathrm{ nm}\right)}{\mathrm{Positive \, control }\left(414 \, \mathrm{ nm}\right)-\mathrm{Negative \, control }\left(414 \, \mathrm{ nm}\right)} \times 100$$

#### Cell-associated hemolytic activity assay

*S. aureus* ATCC 25923 bacterial solution in MHB was prepared as in the minimum inhibitory concentration (MIC) experiment. The bacterial solution was adjusted to 1.5 × 10^6^ CFU/mL at 600 nm using a UV–Vis spectrophotometer, and 5 µL of bacterial solution was added to the round-bottom 96-well plate containing 100 µL of blood mixture in tris-saline (hemolytic activity assay). Serial dilutions of the peptide/conjugate (1024–0.5 µg/mL) prepared in tris-saline with a total volume of 100 µL were added on top of bacteria-blood mix and incubated at 37 °C for 1 h. The bacteria–blood mix and 10% DMSO prepared in Triton X-100 treated with the bacteria–blood mix were used as the negative and positive controls, respectively. All the remaining experimental setups and formulations are identical to those for the hemolytic activity assay.

#### Stability toward plasma proteases

To separate human plasma, EDTA-treated fresh human blood from a healthy donor (Acibadem Mehmet Ali Aydinlar University, Istanbul, Turkey) was centrifuged at 1500 rpm for 5 min, and the supernatant was collected. The peptide and conjugates were mixed with human plasma to a final concentration of 0.1 mg/mL (1.7 µL, 2048 µg/mL sample completed with plasma) and incubated at 37 °C, 1500 rpm in a shaker. Aliquots were collected after 0 h, 6 h, and 24 h incubation and stored at – 20 °C^32^. 5 µL protease inhibitor was added onto 35 µL aliquots.

*S. aureus* ATCC 25923 was cultured in MHA overnight at 37 °C. A colony was then selected and transferred to MHB. The bacterial solution was adjusted to 0.5 McFarland with OD600 value between 0.08 and 0.1 using UV–Vis spectrophotometer and diluted to a 1:200 ratio in MHB. 5 µL of the bacterial suspension was added into serial dilutions of aliquots prepared in MHB (40 µL of total volume) in a round-bottom 96-well plate and incubated overnight at 37 °C. Afterward, 96-well plates were measured at 600 nm using a microplate reader. All samples were prepared in triplicate.

#### Sample preparation for electron microscopy

For scanning electron microscopy (SEM) and transmission electron microscopy (TEM) evaluation, Cpep-PEG-Cpep, pepC-PEG-pepC, TN6 peptide and *E. coli* ATCC 25922 (control group) were studied. Bacteria in the MHB were adjusted to OD600 value between 1.0 using UV–Vis Spectrophotometer. 100 μL (8 × MIC) of each sample and 500 μL of the bacterial solution were mixed and incubated at 37 °C. After 4 h, 10 μL of each sample solution was taken and dropped onto a dialysis membrane (0.45 µm pore size, Spectra/Por) to prepare the SEM samples. All remaining sample solutions were used for the preparation of TEM samples.

#### SEM

The dialysis membrane–containing sample was fixed in 2.5% glutaraldehyde fixative in a Phosphate-buffered saline (PBS, pH 7.2, Sigma-Aldrich) for 4 h. The membrane was washed with the PBS buffer for 12 h. Afterward, postfixation was performed with 1% osmium tetroxide for 1 h and washed again with the PBS for 15 min. The membrane was dehydrated by passing through series of ethyl alcohol concentrations (50, 70, 90, 96 and 100%) for various time points (5, 10, 10, 10, 15 min, respectively). After dehydration, the membranes were rinsed with 2/1, 1/1, 1/2 ethyl alcohol/amyl acetate and pure amyl acetate series for 15 min in each step. The membranes were dried and taken onto grids. Then they were coated with gold after drying in a critical-point dryer and examined using SEM (Quattro SEM, Thermo Scientific)^33^.

#### TEM

The remaining sample solutions from the SEM were centrifuged at 10,000 rpm for 5 min. The supernatant was discarded, and pellets were fixed in 2.5% glutaraldehyde fixative in the PBS buffer (pH 7.2) for 4–12 h. The pellets were washed with the PBS buffer for 5 min and removed and then treated with 1% osmium tetroxide (Sigma-Aldrich) for 1 h. After treatment, the osmium solution was removed and centrifuged at 10,000 rpm for 5 min. After the supernatant was discarded, the pellets were embedded in agar. The samples were washed with 50, 70, 90, 96, and 100% ethyl alcohol series and pure propylene for different times (5, 10, 10, 10, 15 (twice) and 15 min (twice), respectively). Then, 1/1 propylene-Epon was added to the samples, and the samples in the Eppendorf were rotated with a 360 °C tube rotator overnight at room temperature. The agar samples were embedded into pure Epon812 (Sigma-Aldrich) and dried under vacuum for 1 h and outside for 2 h to remove air bubbles. Before taking thin sections (around 60 nm) with Ultramicrotome (EM UC7, Leica) from the agar blocks, the samples were polymerized overnight in an oven at 60 °C. The sections were placed on TEM grids and contrasted. For the contrast step, 1 mL 3% uranyl acetate solution (Sigma-Aldrich) was centrifuged at 14,000 rpm, 4 °C for 15 min, and the supernatant was dropped onto the matte surface of the grid for 25 min. Each grid was then rinsed with distilled water and examined using TEM (Talos L120C, Thermo Scientific)^33^.

#### Statistics

All data were analyzed using GraphPad Prism 9. The results were presented as mean ± SD. Statistical analyses between doses and groups were expressed using two-way analysis of variance (ANOVA) with mixed-effects analysis. A p-value less than 0.05 was considered statistically significant.

### Ethics approval

This study was approved by Acibadem Mehmet Ali Aydinlar University Medical Research Ethics Committee (ATADEK) (Ethics Committee decision no. ATADEK 2023-2/32).

## Supplementary Information

Supplementary Information.

## Data Availability

All data generated or analyzed during this study are included in this published article [and its supplementary information files].
