# Supplementary material for: In vitro efficacy of different PEGylation designs on cathelicidin-like peptide with high antibacterial and antifungal activity
Source: Sci Rep. 2023 Jul 11;13:11213. doi: 10.1038/s41598-023-38449-3 (PMC10336128; doi:10.1038/s41598-023-38449-3)
Supplement: Supplementary file 1 — Supplementary Information. [file 41598_2023_38449_MOESM1_ESM.pdf]

A

Table 1. Physical properties of synthesized modified peptides

Modified Peptide	Modified Peptide Sequence	pI	Net charge at pH 7	Mass (Da)	Hydrophilic Residues (%)
C-TN6	CRLLRLLLRLLR	12.97	4.95	1537.04	33%
TN6-C	RLLRLLLRLLRC	12.97	4.95	1537.04	33%

B

Table 2. Mass to charge ratio of synthesized modified peptides

C-TN6 or TN6-C peptide		
m	z	m/z
1537	1	1537
1537	2	768.5
1537	3	512.3
1537	4	384.3
1537	5	307.4

C

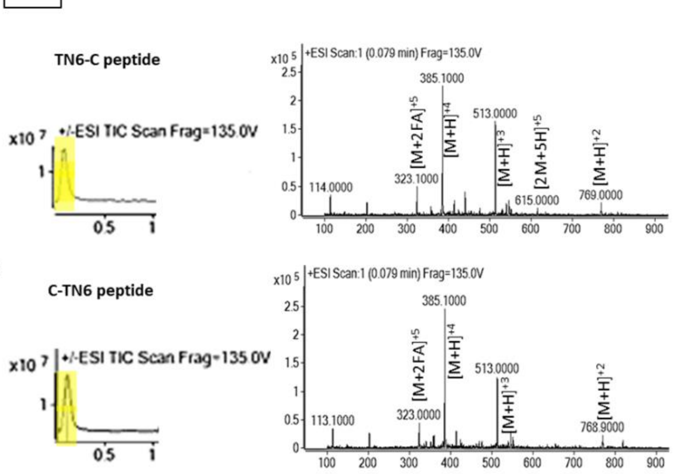

D

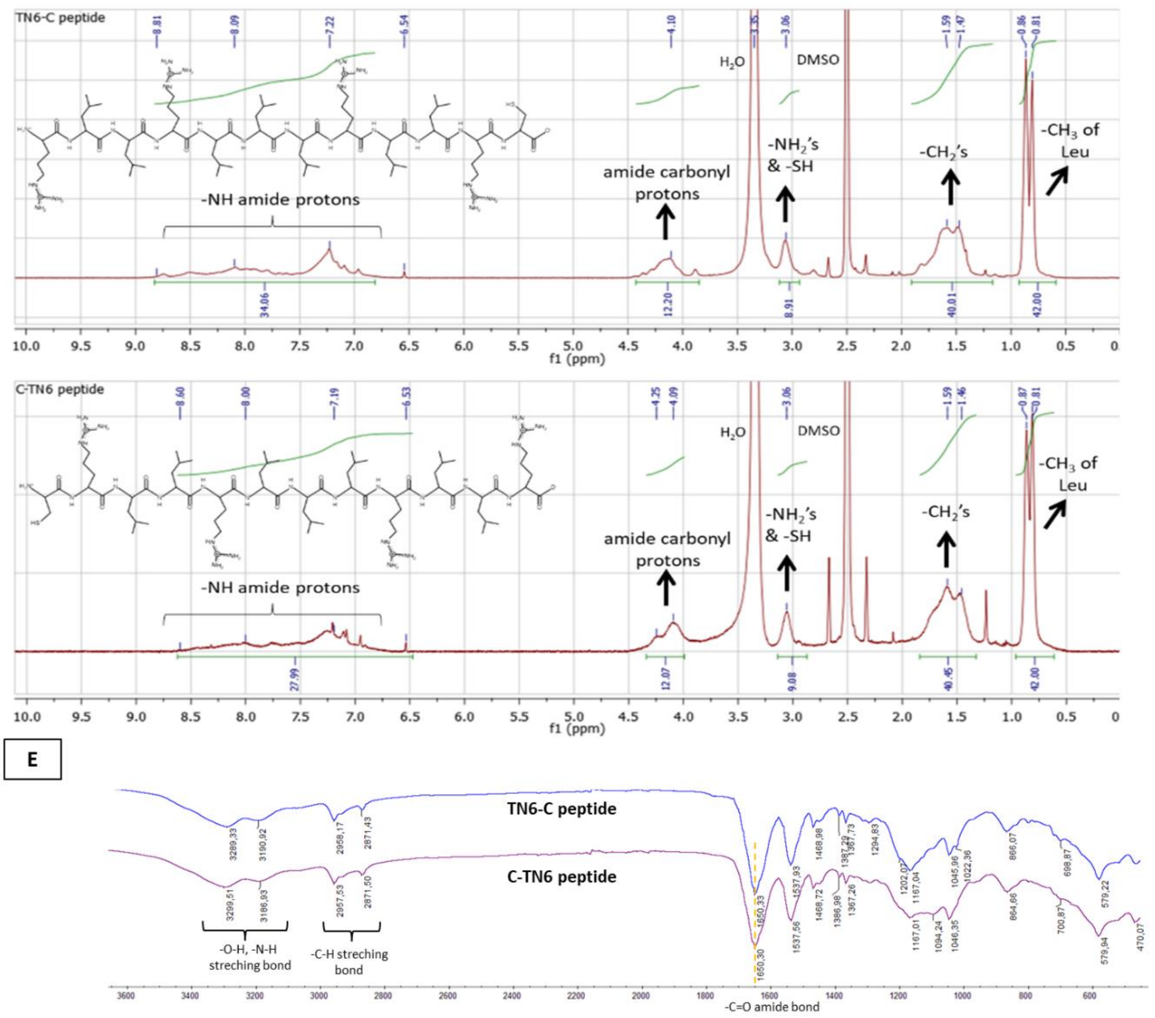

Figure 1 Synthesis and characterization of C-TN6 and TN6-C peptides. A Physical properties of the peptides as sequence, pI, net charge, mass, and hydrophilic residues. B Mass-to-charge ratio (m/z) of the peptides for calculating fragmentation on the MS diagram. C LC-MS/MS results showing possible fragments by analyzing the molecular weight of the peptides. D 1H NMR profile revealing the characteristic protons of the peptide. E FT-IR profile of peptides to confirm intermolecular bonding.

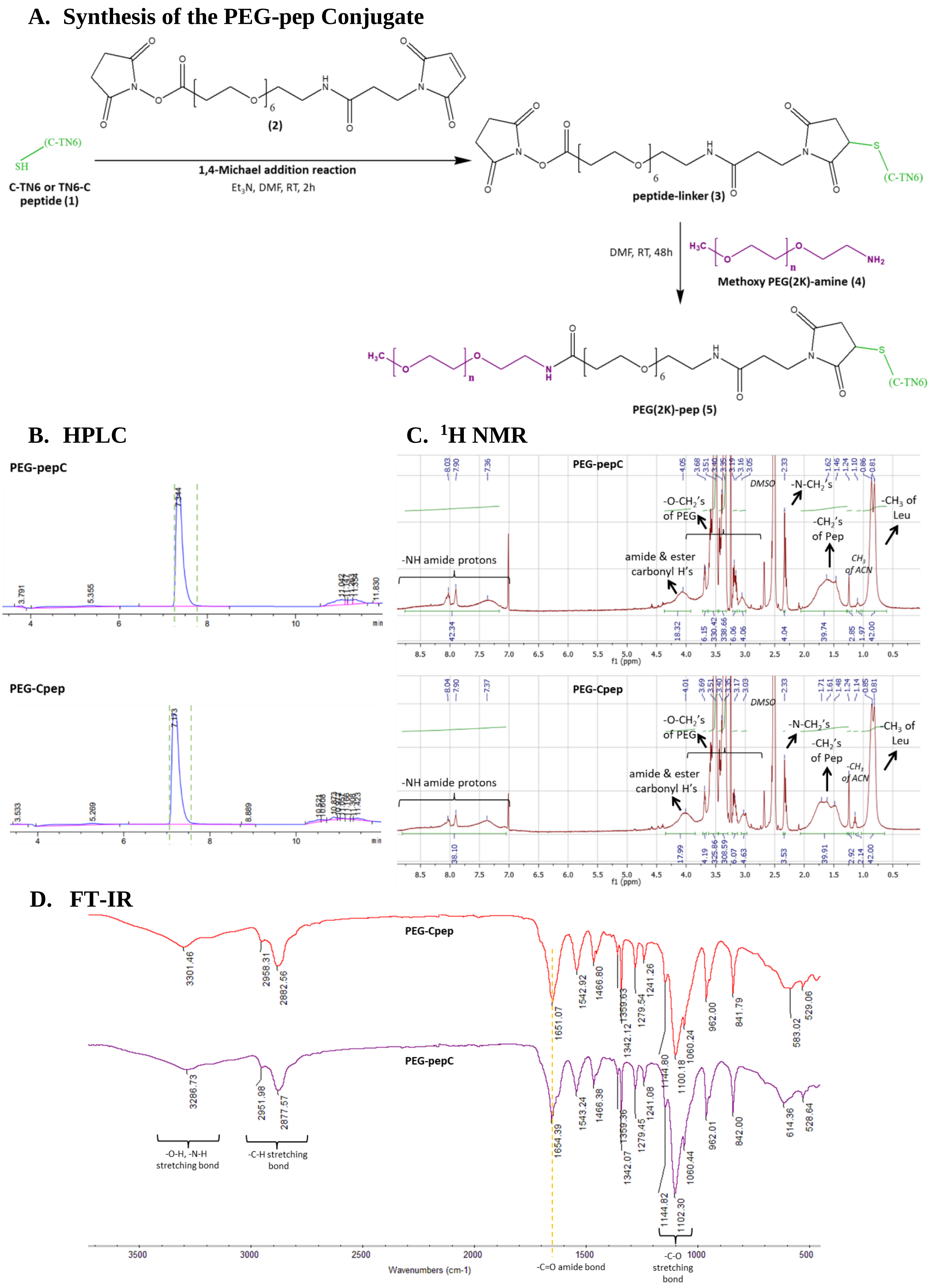

Figure 2. Synthesis and characterization of PEG-pep conjugates. **A** Schematic illustration of stepwise synthesis of PEG-pep conjugates. **B** HPLC chromatograms of PEG-pep conjugates. **C** ^{1}H NMR profile of the PEG-pep conjugates revealing the characteristic protons from the peptide and PEG molecule. **D** FT-IR profile of PEG-pep conjugates to confirm intermolecular bonding.

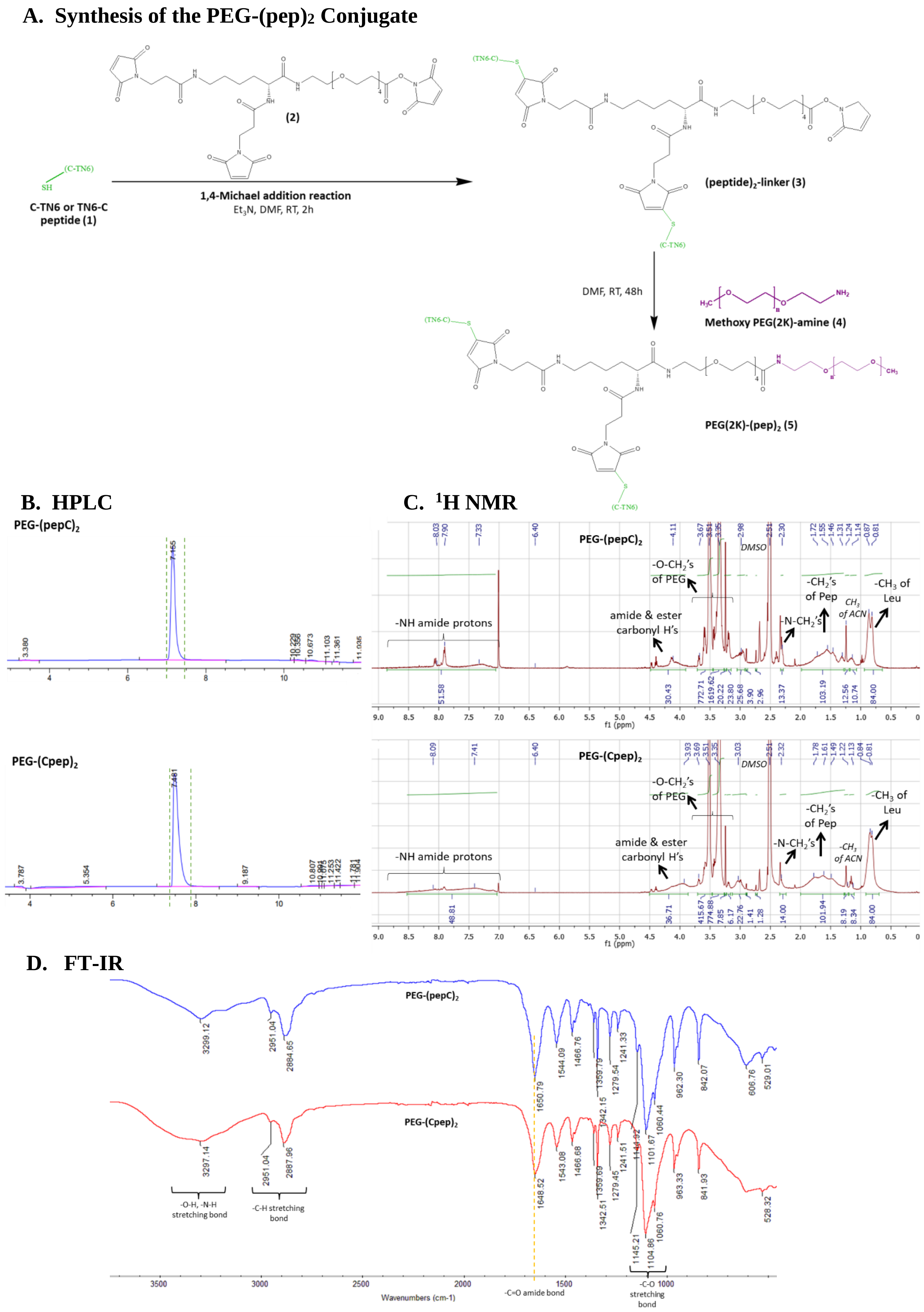

Figure 3 Synthesis and characterization of PEG-(pep)$_2$ conjugates. **A** Schematic illustration of stepwise synthesis of PEG-(pep)$_2$ conjugates. **B** HPLC chromatograms of PEG-(pep)$_2$ conjugates. **C** ^{1}H NMR profile of the PEG-(pep)$_2$ conjugates revealing the characteristic protons from the peptide and PEG molecule. **D** FT-IR profile of PEG-(pep)$_2$ conjugates to confirm intermolecular bonding.
